# Stress among Resident Doctors Working in Different Hospitals of Nepal in the Face of COVID-19 Pandemic: A Descriptive Cross-sectional Study

**DOI:** 10.31729/jnma.5516

**Published:** 2021-06-30

**Authors:** Gauri Adhikari, Leela Paudel, Bidushi Pokhrel, Ganesh Bhandari, Kshitij Kumar Shrestha

**Affiliations:** 1 Hospital for Advanced Medicine and Surgery, Dhumbarahi, Kathmandu, Nepal; 2 Department of Community Medicine, Nepalese Army Institute of Health Sciences, Sanobharyang, Kathmandu, Nepal

**Keywords:** *COVID-19*, *Nepal*, *resident*, *stress*, *stressor.*

## Abstract

**Introduction:**

The emergence and propagation of COVID-19 pandemic has subjected resident doctors to greater workload and consequent psychological implications. Many studies have illustrated various degrees of mental health issues among health care workers in general; however very limited ones have focused primarily on the resident doctors. Therefore, this study aimed to find out the prevalence of stress among the resident doctors of Nepal.

**Methods:**

A descriptive cross-sectional study was carried out in all the teaching hospitals of Nepal with ethical clearance from the Institutional Review Committee (Reference number-245). An online self-designed structured questionnaire developed using Google forms along with questions from stress subscale of Depression, Anxiety and Stress Scale - 21 was disseminated to the residents via social media platforms using Convenience sampling technique. Responses generated were analyzed with Statistical Package for the Social Sciences. Point estimate at 95% Confidence Interval was calculated along with frequency and proportion for binary data.

**Results:**

The prevalence of stress among resident doctors was found to be 16 (8.2%) (4.3-12.1 at 95% Confidence Interval). Greater prevalence of stress was seen among residents working outside Kathmandu valley, those in the frontline and those who were unmarried. Loss of collaborative study/ professional and academic growth experiences was responsible for causing extremely severe stress among 60 (30.9%) residents, followed by stress due to uncertainty regarding COVID-19 58 (29.9%) and unavailability/lack of quality control of personal protective equipment 58 (29.9%).

**Conclusions:**

This study has shed light upon the prevalence of stress and its precipitating factors in Nepalese resident doctors due to COVID-19 pandemic. Our findings could help address these issues for their mitigation promptly.

## INTRODUCTION

The COVID-19 pandemic, since its emergence in December, 2019,^1^ has inundated hospitals around the world with sick patients, putting relentless work pressure and mental health tolls on the Health Care Workers (HCW).^2-7^

At least 3000 HCWs around the world have died because of COVID-19 and this figure is deemed to be highly underreported.^8^ The scenario is akin in Nepal, where HCWs are working with limited resources and still giving their best to care for the ailing patients.

Amongst them, resident doctors pursuing their postgraduate studies in different teaching hospitals in Nepal are more prone to developing health conditions like anxiety, depression and burnout syndromes^9,10^ as they are working in clinical settings for longer durations and taking responsibility for multiple units of a hospital.

This study has attempted to find out the prevalence of stress and the major factors responsible for causing stress among the residents.

## METHODS

A descriptive cross-sectional study was conducted among residents of all the specialties including basic sciences and clinical sciences of 14 Medical College Teaching Hospitals from July 2020 to September 2020. Ethical approval was obtained from Institutional Review Committee (IRC) of Nepalese Army Institute of Health Sciences (NAIHS) (Reference number-245). All the residents of all the specialties including basic sciences and clinical sciences were the study population. Only the residents who gave their consent to participate were included. Convenience sampling technique was used and the sample size was calculated by the formula,

$$\begin{array}{ll} \text{n} & \text{= }{\text{Z}}^{\text{2}}\times \text{p}\times \text{(1}-\text{p)}/{\text{e}}^{\text{2}}\\ & \text{= }{\left(1.96\right)}^{\text{2}}\times 0.294\times 0.706/{(\text{0.07)}}^{\text{2}}\\ & \text{= }163 \end{array}$$

Where,

n = sample size,
Z = 1.96 at 95% Confidence Interval (CI)
p = prevalence of stress in Physician Trainees, 29.4%^11^
e = margin of error, 7%.

However, the total responses included in this study were 194.

For the assessment of stress level questions pertaining to stress were extracted from the Depression Anxiety Stress Scales-21 (DASS-21) scoring system.^12,13^ To assess the stress factors, a self-designed questionnaire was used and validated by experts. It was then pretested among 20 resident doctors.

For data collection an online questionnaire generated by Google forms and disseminated among the participants with the use of applications like Email, Viber, Whatsapp and Facebook was done. Responses collected from Google form were analyzed using IBM Statistical Package for Social Sciences version 22.0 for Windows. Descriptive statistics were used and variables were represented in terms of numbers and percentages.

## RESULTS

Out of 194 participants, the prevalence of stress was seen in 16 (8.2%) (4.3-12.1 at 95% CI) participants as definable by the DASS-21 questionnaire. 144 (74.2%) were between age group of 25-30 years, 50 (25.8%) were 30 years and above. 156 (80.4%) of them were working as frontline health workers (Table 1).

**Table 1:** Socio-demographic distribution of the participants.

Variables		n (%)
Age	25-30	144 (74.2)
	30-35	46 (23.7)
	≥36	4(2.1)
Gender	Male	100 (51.5)
	Female	94 (48.5)
Marital status	Married	116 (59.8)
	Unmarried	78 (40.2)
Working as frontliner	Yes	156 (80.4)
	No	38 (19.6)
Working hour	Less than 48	34 (17.5)
	48-72	64 (33.0)
	More than 72	96 (49.5)
PCR test	Done	60 (30.9)
	Not done	134 (69.1)
Test result out of those tested	Negative	48 (24.7)
	Prefer not to say	12 (6.2)

It was observed that among the 68 residents working outside the Kathmandu valley, 8 (11.8%) of them were stressed compared to only 8 (6.5%) among the 118 working within the valley. Furthermore, more number of participants working in the frontline reported being stressed 14 (9%) than those not working in the frontline 2 (5.3%). The prevalence of stress was more in participants aged less than 30 years 10 (9.1%) compared to those of ages 30 and above 6 (7.1%). There were no differences in stress levels experienced according to gender and variability in working hours. Respondents who were unmarried had greater prevalence of stress 8 (10.3%) than those who were married 8 (6.9%) (Table 2).

**Table 2:** Stress distribution among different socio-demographic variables.

Variables		Stress	
		Yes n (%)	No n (%)
Age	Less than 30	10 (9.1)	100 (90.9)
	30 and above	6 (7.1)	78 (92.9)
Gender	Female	8 (8.5)	86 (91.5)
	Male	8 (8.0)	92 (92.0)
Marital	Married	8 (6.9)	108 (93.1)
status	Unmarried	8 (10.3)	70 (89.7)
Front Liner	Yes	14 (9.0)	142 (91.0)
	No	2 (5.3)	36 (94.7)
Working Hours	More Than 72 Hours	8 (8.3)	88 (91.7)
	Less than 72 hours	8 (8.2)	90 (91.8)
Working	Outside Valley	8 (11.8)	60 (88.2)
Area	Inside valley	8 (6.5)	118 (93.7)

Furthermore, among the stressors, loss of collaborative study and professional/academic growth experiences was one of the most important, causing extremely severe stress in 60 (30.9%). This was followed by the uncertainty regarding the situation of COVID-19 with 62 (32%) and 58 (29.9%) facing severe and extremely severe stress respectively. The stress of being infected while working in frontline had been causing moderate stress in 72 (37.1%). Stress due to the risks of transmitting infection to one's family members was equivocally rated as being severe and very severe by 50 (25.8%) participants each. Unavailability/ lack of quality control of personnel protective equipment (PPE) was reported to cause very severe stress among 58 (29.9%) respondents. 66 (34%) participants had severe levels of stress due to inadequacy/ unavailability of PCR testing for the HCWs and general population. Separation from family during long working hours caused moderate, severe and very severe stress among 46 (23.7%), 40 (20.6%) and 46 (23.7%) participants respectively. 64 (33%) residents reported facing moderate levels of stress due to feeling of failure in the face of poor prognosis of COVID-19 patients. Stress due to not being able to collaborate with higher authorities for putting forward the problems faced by self was reported to be moderate by the highest number of respondents 80 (41.2%). Also, 45 (23.2%), 54 (27.8%) and 44 (22.7%) respondents respectively mentioned moderate, severe and very severe levels of stress due to lack of well-equipped rooms for isolating COVID-19 patients in their hospitals. Lack of incentives and pay cuts during the COVID-19 pandemic was causing extremely severe, severe and moderate stress among 50 (25.8%), 46 (23.7%) and 44 (22.7%) respondents respectively. Stress due to decline in health care response towards other diseases apart from COVID-19 caused moderate stress among the maximum number of participants 72 (37.1%) while causing severe stress in 50 (25.8%). Social stigma related to COVID-19 was found to have no stress among 30 (15.5%) participants while causing severe stress among 52 (26.8%) of them (Table 3).

**Table 3:** Level of stress caused by various stressors.

S.N	Stressors	Level of stress n (%)				
		Normal	Mild	Moderate	Severe	Extremely severe
1	Infected	16 (8.2)	38 (19.6)	72 (37.1)	42 (21.6)	26 (13.4)
2	Transmission of the infection to one's family	20 (10.3)	16 (8.2)	44 (22.7)	50 (25.8)	50 (25.8)
3	Unavailability and quality control of personal protective equipment (PPE)	18 (9.3)	20 (10.3)	48 (24.7	50 (25.8)	58 (29.9)
4	Inadequacy /unavailability of PCR testing for the health workers and general population	24 (12.4)	20 (10.3)	48 (24.7)	66 (34.0)	36 (18.6)
5	Separation from family during long working hours in the hospital	28 (14.4)	34 (17.5)	46 (23.7)	40 (20.6)	46 (23.7)
6	Feeling of failure in the face of poor prognosis of COVID-19 patient	16 (8.2)	40 (20.6)	64 (33.0)	44 (22.7)	30 (15.5)
7	Not being able to collaborate with higher authorities to express the problems faced by self	12 (6.2)	32 (16.5)	80 (41.2)	40 (20.6)	30 (15.5)
8	Lack of proper and well-equipped rooms for isolating COVID-positive patient	16 (8.2)	35 (18.0)	45 (23.2)	54 (27.8)	44 (22.7)
9	Pay cuts/lack of incentives during the time of this pandemic	26 (13.4)	28 (14.4)	44 (22.7)	46 (23.7)	50 (25.8)
10	Loss of collaborative study /professional and academic growth experiences	10 (5.2)	22 (11.3)	42 (21.6)	60 (30.9)	60 (30.9)
11	Decline in health care response towards other diseases	14 (7.2)	26 (13.4)	72 (37.1)	50 (25.8)	32 (16.5)
12	Uncertainty regarding COVID-19	4(2.1)	24 (12.4)	46 (23.7)	62 (32.0)	58 (29.9)
13	Social stigma (accused of being a carrier, asked to leave a rented place) related to being a medical professional)	30 (15.5)	30 (15.5)	46 (23.7)	52 (26.8)	36 (18.6)

by Lai et al, symptoms of distress were seen in 71.5% of HCWs, with more severe degrees of distress in the

## DISCUSSION

This study presents our assessment regarding the subjective burden of stress of Nepalese resident doctors during COVID-19 pandemic. Our study has shown the prevalence of stress in resident doctors to be 8.2% which is considerably less in comparison to other similar studies around the globe. The metaanalysis conducted by Salari et al showed the prevalence of stress to be 29.6%.^14^ In another study frontline HCWs.^15^

Stress prevalence was not seen to differ among demographic variables like age, gender and marital status of the participants. 14 (87.5%) of resident doctors who experienced stress were front liners. It was possibly because of increased burden of patient care and risk of exposure to infection. Likewise, a greater number of participants working in hospitals outside the Kathmandu valley (11.8% compared to 6.5% working inside the valley) reported being stressed probably due to the lack of attention towards health care demands in the sub-urban and remote areas, inadequate manpower and insufficient protection to the HCWs from violence^16^ to name few. Contrary to what would generally be expected, working hours (<72 or >72) didn't make much difference between the stress outcomes in participants, with nearly equal prevalence of stress in these groups.

Our study shows that out of the 13 stress factors that were included in the questionnaire, loss of collaborative study, professional and academic growth experiences caused the most profound level of stress among residents (extremely severe in 30.9%). Residency is a formative time in training based upon the principles of graduated responsibility and autonomy.^17^ The future excellence of residents depends largely on collaborative study experiences like research, conferences and group interactions,^18^ and this aspect of their training has been severely hampered due to the nation-wide lockdown of four months.^19^ Both unavailability/ lack of quality control in PPE and uncertainty regarding COVID-19 have caused extremely severe stress to 29.9% of the residents. Since PPE has not been adequately supplied ^20,21^ and quality check has not been done effectively,^22^ the fear of contracting infection has constantly distressed the residents. Even in studies performed in the USA, the availability of PPE for HCWs is a tremendously debated issue and in many countries, the lack of PPE has been defined as a source of anxiety among HCWs.^22-23^ Furthermore, the disease pattern of SARS-COV-2 is yet to be established, leading to the inevitable anxiety among HCWs around the world. In comparison to the foreign countries, sadly Nepal has not been able to provide adequate PCR testing even for the HCWs themselves,^24^ leading to severe stress as observed in our study in 34% participants. This imposes a higher risk of transmission of infection to large number of patients being treated by these residents who might have asymptomatically been harboring the infection.

Contrary to the huge existence of social stigma associated with COVID-19 being reported by newspapers frequently,^25^ our study has shown the least amount of stress (no stress mentioned by 15.5% respondents) in this regard, which is possibly because they have experienced these kinds of mistreatments on a daily basis. In a study performed in Nepal, more than half of HCWs (53.7%) mentioned facing stigma due to COVID-19, one-fourth of them (26.7%) being stigmatized because of their profession, 21.5% accused of being a carrier, 3.2% threatened and 2.3% being asked to leave a rented place.^10^ Moreover, in a country like ours where insufficient economic aid is given to health sector, lack of incentives and unexpected pay cuts^26^ in this health crisis has been demotivating the HCWs and making it difficult to make their ends meet, demonstrated by the prevalence of at least some level of stress among 91.8% of the participants.

Surprisingly, it was observed that nearly twice as more residents were stressed regarding transmission of COVID to their family members than contracting the infection themselves, probably owing to the kind, giving nature of the profession they have delved into and always putting others in priority. In a similar study conducted on surgery residents, they overwhelmingly reported "risk to loved ones" (84% of respondents) as the most anxiety-provoking aspect of their potential contraction of COVID-19, compared to "Risk to self" (47% of respondents).^27^

Our study has some limitations. Firstly, it was an online survey, limiting face-to-face interaction with the participants, creating cognitive bias. Only resident doctors were taken as study units, therefore limiting study sample and not including control groups. The use of self-designed questionnaire was not able to include all the possible variables, decreasing accuracy of the study. Since the study was not funded by any external sources, it couldn't be conducted extensively. The travel limitations during nation-wide lockdown limited effective collaboration with respective teaching hospitals and thus limiting the number of participants.

## CONCLUSIONS

To conclude, this is one of the few studies of its kind in Nepal where the prevalence of stress and stress factors experienced by resident doctors have been pointed out. The prevalence of stress was found to be much higher in frontline residents, especially in those who were working outside Kathmandu valley. Furthermore, loss of collaborative studies and professional/ academic growth experiences was found to be causing the most severe form of stress in the maximum number of residents followed by unavailability/ lack of quality control in PPE and the uncertainty regarding the nature of COVID-19. Since the residents are working in all the provinces of Nepal and dedicating most of their time in patient care, they can be taken as the representative units of health manpower. Hence, measures inculcated to alleviate their stress and address their problems could benefit all the other HCWs. This would consequently lead to better health care delivery to patients and overall mitigation of the health crisis in the face of this deadly pandemic.
